# Abscisic Acid-Induced Stomatal Closure: An Important Component of Plant Defense Against Abiotic and Biotic Stress

**DOI:** 10.3389/fpls.2021.615114

**Published:** 2021-03-04

**Authors:** Pulimamidi Bharath, Shashibhushan Gahir, Agepati S. Raghavendra

**Affiliations:** Department of Plant Sciences, School of Life Sciences, University of Hyderabad, Hyderabad, India

**Keywords:** pathogen resistance, water use, stress adaptation, guard cells, signaling components

## Abstract

Abscisic acid (ABA) is a stress hormone that accumulates under different abiotic and biotic stresses. A typical effect of ABA on leaves is to reduce transpirational water loss by closing stomata and parallelly defend against microbes by restricting their entry through stomatal pores. ABA can also promote the accumulation of polyamines, sphingolipids, and even proline. Stomatal closure by compounds other than ABA also helps plant defense against both abiotic and biotic stress factors. Further, ABA can interact with other hormones, such as methyl jasmonate (MJ) and salicylic acid (SA). Such cross-talk can be an additional factor in plant adaptations against environmental stresses and microbial pathogens. The present review highlights the recent progress in understanding ABA’s multifaceted role under stress conditions, particularly stomatal closure. We point out the importance of reactive oxygen species (ROS), reactive carbonyl species (RCS), nitric oxide (NO), and Ca^2+^ in guard cells as key signaling components during the ABA-mediated short-term plant defense reactions. The rise in ROS, RCS, NO, and intracellular Ca^2+^ triggered by ABA can promote additional events involved in long-term adaptive measures, including gene expression, accumulation of compatible solutes to protect the cell, hypersensitive response (HR), and programmed cell death (PCD). Several pathogens can counteract and try to reopen stomata. Similarly, pathogens attempt to trigger PCD of host tissue to their benefit. Yet, ABA-induced effects independent of stomatal closure can delay the pathogen spread and infection within leaves. Stomatal closure and other ABA influences can be among the early steps of defense and a crucial component of plants’ innate immunity response. Stomatal guard cells are quite sensitive to environmental stress and are considered good model systems for signal transduction studies. Further research on the ABA-induced stomatal closure mechanism can help us design strategies for plant/crop adaptations to stress.

## Introduction: ABA and Plant Defense

Plants need to respond quickly to diverse stress conditions, as they cannot move away. Stress can be due to abiotic (e.g., drought, salinity, chilling, and high-temperature) or biotic factors (e.g., pathogens, insects, herbivores) (Zhu, 2016; Lamers et al., 2020). Plants developed various adaptation strategies to cope up with these situations. A typical example is the stomatal closure, limiting the water loss and restricting pathogen entry into the leaves (Melotto et al., 2008; Gudesblat et al., 2009a; Sussmilch and McAdam, 2017; Agurla et al., 2018a). Plants accumulate hormones [e.g., abscisic acid (ABA) or salicylic acid (SA) or methyl jasmonate (MJ)] under abiotic stress conditions and elicitors [e.g., flagellin 22 (flg22)] under pathogen attack. Among the hormones, ABA is involved in several abiotic and biotic stress conditions and is therefore considered an essential and versatile compound. In contrast, SA, MJ, and ethylene (ET) help in resistance against biotic stress. Under drought, salinity, or cold stress, ABA accumulation causes stomatal closure to conserve water while up-regulating genes to promote osmotic adjustment in leaves (Lim et al., 2015; Zhao et al., 2017; Niu et al., 2018). The enhanced ABA levels in plants mediate the cross-adaptation against drought and pathogens besides insect herbivores (Lee and Luan, 2012; Nguyen et al., 2016).

Several compounds other than ABA also accumulated in plants in response to different stresses (Table 1). These compounds can close stomata and, in many instances, improve plants’ resistance to pathogens. The plant hormones and elicitors can further regulate transcription factors and induce pathogenesis-related (PR) genes (Bielach et al., 2017; Breen et al., 2017). There can also be a cross-talk between the factors involved in abiotic and biotic stress signaling (Nejat and Mantri, 2017; Saijo and Loo, 2020). However, these compounds either require ABA for their action or interact with ABA to activate defense responses.

**TABLE 1:** A spectrum of compounds that accumulate in plant cells along with ABA during biotic/abiotic stress and can promote stomatal closure.

Hormone/compound	Type of stress	References	Reason of closure
ABA	Drought, cold, salinity and heat	Nakashima et al., 2014	Increased ABA levels
Allyl isothiocyanate (AITC)	Wounding, insect, herbivore (biotic)	Khokon M.A. et al., 2011	Production of ROS and NO. elevated Ca^2+^ levels
Ethylene (ET)	Drought, ozone	Wilkinson and Davies, 2010	Mediated H_2_O_2_ production in ABA signaling
Hydrogen sulfide (H_2_S)	Drought	Jin et al., 2013	H_2_S affected ABA responses and ABA increased H_2_S levels
Inositol 1,4,5-trisphosphate (IP_3_)	Drought and salt stress	Jia et al., 2019	Stimulated Ca^2+^ release in the cell and ABA responses
Methyl jasmonate (MJ)	Wounding (biotic)	Förster et al., 2019	Signaling events overlap with ABA action
	Pathogen, insects (biotic)	Verma et al., 2016	Marked interaction with ABA and SA
Phosphatidic acid (PA)	Heavy metal (Arsenic) stress	Armendariz et al., 2016	Induced accumulation of PLD and PA, are due to ABA
Polyamines (PAs)	Drought	Adamipour et al., 2020	ROS and NO production. ABA caused accumulation of PAs
	PEG-induced osmotic stress, chilling	Pál et al., 2018	Increased PAs stimulated ABA accumulation
Proline and G-substances	Drought	Raghavendra and Reddy, 1987	Decreased proton efflux and K^+^ content, as in case of ABA
Salicylic acid (SA)	Bacterial invasion (biotic)	Melotto et al., 2006	SA-action overlapped with ABA signaling
Sphingosine-1-phosphate (S1P)	Drought	Ng et al., 2001	Mobilized Ca^2+^ and mediated stomatal closure by ABA
Strigolactone (SL)	Drought and salt stress	Ha et al., 2014	ABA and SL cross-talk positively regulated stomatal closure
Sulfate (in xylem sap)	Drought	Malcheska et al., 2017	Promoted ABA synthesis in guard cell

*The compounds are arranged in alphabetical order. The abbreviations are listed in the Appendix (last page).*

Since its discovery, studies on ABA (a sesquiterpene) and its role in plant processes were studied extensively. Plant processes such as seed dormancy, seed development, promotion of desiccation tolerance, abscission, and, most importantly, stomatal closure were all regulated by ABA (Lim et al., 2015). Further, ABA can be crucial in also non-stress conditions (Yoshida et al., 2019). The action of ABA was complemented by hormones, such as SA (Robert-Seilaniantz et al., 2011; Wang H.Q. et al., 2020). Similarly, some of the secondary messengers triggered by ABA can also participate in plants’ adaptation to abiotic and biotic stress. Examples are reactive oxygen species (ROS), nitric oxide (NO), and cytosolic free Ca^2+^ (León et al., 2014; Huang et al., 2019). Several compounds like polyamines (PAs), hydrogen sulfide (H_2_S), and brassinosteroids (BRs) promote drought tolerance by regulating ABA synthesis and *vice versa* (Jin et al., 2013; Ha et al., 2014; Adamipour et al., 2020).

Readers interested in ABA and its role in plants may refer to some recent reviews (Kumar et al., 2019; Chen K. et al., 2020; Gietler et al., 2020; McAdam and Sussmilch, 2020). Our review emphasizes the role of ABA’s stomatal closure as an adaptive measure against both abiotic and biotic stresses. We have also discussed other compounds that can improve plant adaptations, still involving ABA. The stomatal closure by ABA follows a typical scheme of signal transduction. The interactions of these signaling components with others to synergize the plant’s adaptation against pathogen attacks are described. To limit the length of our article, reviews were cited when available. In some instances, original articles were referred, due to their classic importance.

## Increased ABA Levels Under Different Stress Conditions

When plants were exposed to water stress (drought), an increase in ABA was typical due to either synthesis or degradation of ABA or both (Ma et al., 2018; Chen K. et al., 2020; Gietler et al., 2020). The soil-water deficit could be perceived as a signal by roots to trigger ABA’s *de novo* synthesis (Jiang and Hartung, 2008; Fang and Xiong, 2015; Qi et al., 2018). The increase in ABA of roots in response to drought was correlated with an increase in foliar-ABA concentrations, suggesting drought-induced ABA played a significant role in controlling leaf water potential (Zegada-Lizarazu and Monti, 2019). ABA accumulated in roots was transported to trigger stomatal closure in leaves and limit transpirational water-loss (Haworth et al., 2018). An increase in ABA could also occur in response to temperature-stress (high or low) (Tao et al., 2016; Karimi, 2019) or a newly discovered small peptide, CLE25 (Takahashi et al., 2018). Sato et al. (2018) found that NCED3 could be the trigger to enhance ABA biosynthesis in *Arabidopsis* under drought stress. Under these conditions, increased ABA and stomatal closure could limit the water-loss and restrict pathogen entry (Wu et al., 2007; Alazem and Lin, 2015). This phenomenon was complemented with additional steps of ABA transport from roots to shoots, conversion of bound ABA into free form to mobilize ABA within leaf (Hewage et al., 2020; Xylogiannis et al., 2020).

An increase in endogenous levels of ABA was also observed when plants were infected with pathogens, for e.g., *Phaseolus* by *Colletotrichum* (Dunn et al., 1990), flax by *Fusarium* (Boba et al., 2020), and *nced5* mutant of Arabidopsis by *Alternaria* (Fan et al., 2009). Similarly, the clonal variation of chestnut susceptibility or resistance to *Fusarium* was related to ABA levels under infection (Camisón et al., 2019). The exact relationship between endogenous ABA levels and disease susceptibility of plants appeared to be complex, as the relationship depended on the duration of infection, other stresses, and the type of pathogen (Asselbergh et al., 2008). During the early stages of pathogen infection, the increased ABA levels helped in resistance, while at later stages, high levels of ABA made the plants susceptible to pathogens (Maksimov, 2009). The differential effects of ABA on the modulation of pathogen sensitivity need to be examined further, particularly in relation to the predisposition of plant tissue. Readers interested in ABA accumulation mode may refer to relevant articles for further details (Maksimov, 2009; Finkelstein, 2013; Ali et al., 2020; Chen K. et al., 2020).

## Stomatal Closure: A First Line of Defense Against Diverse Stress Conditions

Stomatal closure is one of the initial responses of plants to stress conditions to retain water status and provide innate immunity against pathogens (McLachlan et al., 2014; Arnaud and Hwang, 2015; Agurla et al., 2018a). The physical barriers on the plant’s outer surface, such as bark, cuticle, and cell wall, could protect against physical and biological factors. However, the microscopic pores on leaf surfaces called stomata are the accessible entrances to several microbes. Stomata form the gateways for transpiration, photosynthetic gas exchange as well as microbial entry into leaves. Stomatal guard cells are quite dynamic in sensing and responding to external microbial pathogens. Stomatal closure can be an essential strategy to defend against abiotic and biotic factors such as drought or pathogens (Lim et al., 2015; Melotto et al., 2017; Nejat and Mantri, 2017). Several instances of stomatal closure induced by plant pathogens are listed in Table 2. Stomatal closure was triggered by either elicitors or other compounds produced in the leaf in response to pathogens, such as SA, MJ, or PAs. Stomata can sense and respond to microbe-associated molecular patterns, including chitosan, flagellin, and harpin (Zhang L. et al., 2017; Klessig et al., 2018). The sensing of ABA or other compounds and the final response of stomatal closure follows a common signaling pathway involving receptors, protein kinases, secondary messengers, ion channels, ion efflux, and turgor loss in guard cells. Among kinases, OST1 is a primary activating factor NADPH oxidase and raises the ROS levels of guard cells.

**TABLE 2:** Several compounds that induce stomatal closure can also promote pathogen resistance of plants.

Compound/Hormone	Effect on stomata	References	Response to Pathogen	References
ABA	Closure	Cummins et al., 1971	Increased callose deposition and enhanced resistance against *Leptosphaeria maculans* and *Pseudomonas syringae*	Oide et al., 2013
Allyl isothiocyanate (AITC)	Closure	Khokon M.A. et al., 2011	Required ccoperative MJ-priming to evade pathogens	Khokon M.A. et al., 2011
Cerato-platanin	Closure	Baccelli et al., 2014	Increased ROS levels, and interaction with SA/ethylene for resistance against *Botrytis cinerea* and *P. syringae*	Baccelli et al., 2014
Chitin	Closure	Ye et al., 2020b	Converted to chitosan and caused guard cell death to restrict fungal pathogen invasion	Ye et al., 2020b
Chitosan	Closure	Srivastava et al., 2009	Immunity against *Fusarium* associated with stomatal closure	Narula et al., 2020
Cryptogein	Closure	Gayatri et al., 2017	Produced ROS and induced PCD for resistance against *Hyaloperonospora arabidopsidis*	Kurusu et al., 2018
Cyclodipeptides	Closure	Wu et al., 2017	Increased defense responses to *Phytophthora nicotianae* and *Tobacco mosaic virus*	Wu et al., 2017
Ethylene (ET)	Closure	Desikan et al., 2006	Promoted production of ROS accumulation and phytoalexin to boost resistance against *Magnaporthe oryzae*	Yang et al., 2017
Cytokinin	Closure	Novák et al., 2013	Induced HR-like response, cell death and activation of PR genes, in response to *Agrobacterium tumefaciens*	Novák et al., 2013
Methyl jasmonate (MJ)	Closure	Raghavendra and Reddy, 1987	Interacted with ethylene for resistance against *B. cinerea*	Song et al., 2014
PAMP-induced peptide (PIP1)	Closure	Hou et al., 2014	Stimulation of MAPK, ROS accumulation and callose deposition for resistance against *F. oxysporum* and *P. syringae*	Hou et al., 2014
Salicylic acid (SA)	Closure	Manthe et al., 1992	Promoted synthesis of catechin and proanthocyanidins to defend against *Melampsora larici-populina*	Ullah et al., 2019
Strigolactone (SL)	Closure	Lv et al., 2018	Down-regulated MYC2 and upregulated plant defense factor upon *Meloidogyne incognita* infection	Xu et al., 2019

*Further details of the mechanism of stomatal closure by several of these are indicated in Table 3. The abbreviations are listed in the Appendix (last page).*

**FIGURE 1:**
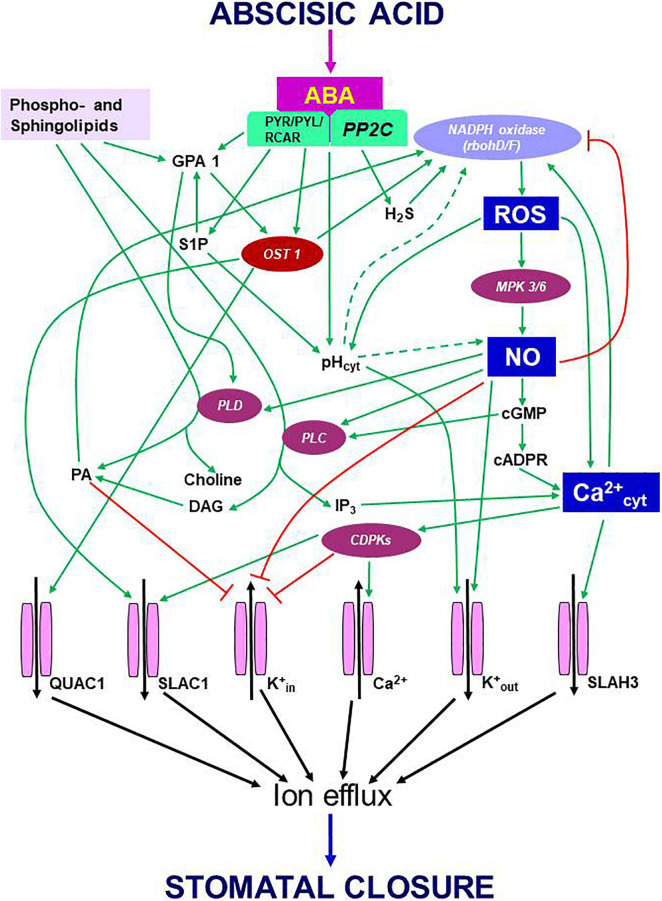
Schematic representation of eventsm during signal transduction pathway induced by ABA leading to stomatal closure. Binding of ABA to its receptor (PYR/PYL/RCAR) blocks the function of PP2C. As a result, OST1, which stays phosphorylated, activates multiple components like NADPH oxidase (to generate ROS) and anion channels [quick anion channel 1 (QUAC1) and slow anion channel 1 (SLAC1)] to trigger anion efflux. The secondary messengers: ROS, NO, and cytosolic Ca^2+^, exert multiple effects. The rise in cytosolic pH, another secondary messenger, appears to stimulate NADPH oxidase, but neither the origin nor the mode of pH action is understood. The high levels of ROS can promote NO production with the involvement of mitogen-activated protein kinases and elevate pH and Ca^2+^ in the cytosol. In turn, Ca^2+^ can activate RBOH-D/F and elevate ROS levels. The rise in NO downregulates K^+^ inward channels and elevates cytosolic Ca^2+^ levels through cyclic guanosine monophosphate (cGMP) and cyclic ADP ribose (cADPR). An increase in Ca^2+^ can activate calcium-dependent protein kinases to facilitate a further influx of Ca^2+^ from outside. Ca^2+^-activated calcium-dependent protein kinases stimulate SLAC1 and S-type anion channel 3 while inhibiting K+ influx through K^+^_in_ channels. When present, NO activates two enzymes, phospholipase C and phospholipase D (PLD), resulting in the synthesis of inositol 1,4,5-triphosphate (IP_3_) phosphatidic acid. In turn, IP_3_ releases Ca^2+^ levels from internal stores of plant cells, while phosphatidic acid can stimulate NADPH oxidase and inhibit the inward K^+^ channel. ABA can stimulate the formation of sphingosine 1-phosphate (S1P) and phytosphingosine-1P, which activate PLD through G-protein α-subunit 1 (GPA1). NO can promote K^+^ efflux channels and cytosolic alkalization while inhibiting K^+^ influx channels via calcium-dependent protein kinases. These three secondary messengers involved in ABA signaling, namely ROS, NO, Ca^2+,^ and their interactions, play a significant role in regulating stomatal closure. Ion channels are terminal points of signal transduction, causing the loss of turgor in guard cells and stomatal closure. Further details are described in the text. Arrows (→) indicate stimulation, and the symbol ⊣ represents inhibition. Abbreviations used here are listed in the Appendix.

During ABA-induced stomatal closure, an increase in OST1 kinase was followed by the activation of RBOH D/F, and increases in ROS/NO/Ca^2+^ levels. In turn, Ca^2+^ dependent CDPKs activated slow anion channel 1 (SLAC1), S-type anion channel 3 (SLAH3) and K^+^out channels to promote ion efflux from guard cells and forced stomata to close. However, in presence of flg22 or yeast elicitor, the activity of OST1 did not increase (Montillet et al., 2013; Ye et al., 2015). Albeit in a resting stage, OST1 participated in stomatal closure by variety of signals including PAMPs (e.g., flg 22, yeast elicitor, chitosan) or environmental components, such as high CO_2_ or high humidity (Melotto et al., 2006; Ye et al., 2015, 2020b; Ye and Murata, 2016; Pantin and Blatt, 2018). Besides its action through ROS/NO/Ca^2+^, OST1 could directly modulate ion channels to cause stomatal closure (Figure 1). In a recent study, the events involving OST1/SnRK2s were studied in real-time using FRET sensors (Zhang et al., 2020). These experiments provided a visual evidence of the interaction of OST1 with signaling components of ABA and elevated CO_2_. It is obvious that OST1 is an important point of convergence of signals from abiotic and biotic factors. It would be interesting to assess the mechanism by which OST1 keeps up such dual mode of activation and modulating downstream components, all converging to mediate stomatal closure.

There are reports indicating that stomatal closure can be induced by biotic and abiotic stresses in an “OST1-independent manner” (Hsu et al., 2018; Zheng et al., 2018). For e.g., plant elicitor peptides (Peps), a group of damage-associated molecular patterns, can trigger stomatal closure by activating SLAC1 and SLAH3 in an OST1-independent manner (Zheng et al., 2018). Similarly, elevated CO_2_ can bypass OST1 kinase and activate SLAC1 (Hsu et al., 2018). SLACs may be activated without the involvement of OST1 but other kinases. For e.g., the signaling events in guard cells can utilize MAPK cascade to up-regulate SLAC1/SLAH3 (Jagodzik et al., 2018). MAP kinases 3/6 participated upstream of NO during stomatal closure in darkness (Zhang T.Y. et al., 2017), while MPK 9/12 activated SLAC1 integrating with Ca^2+^/CDPK system during the cross-talk of ABA and SA (Prodhan et al., 2018), though the exact mechanism is not known. When exposed to a small elicitor peptide, AtPeps, a two-kinase component of BRASSINOSTEROID INSENSITIVE 1-associated receptor kinase 1 (BAK1)/BORTRYTIS-INDUCED KINASE 1 (BIK1) was turned on to activate SLAC1 and SLAH3 in guard cells (Zheng et al., 2018). However, further experiments are necessary to identify the exact components involved in the activation of SLAC1 by MPK 9/12 or BAK1/BIK1.

There can be additional components of a leaf that can provide resistance to microbes as well as environmental stresses, e.g., callose or silicon deposition (Ellinger and Voigt, 2014; Alhousari and Greger, 2018; Islam W. et al., 2020), cuticular waxes (Lewandowska et al., 2020) and trichomes (Fürstenberg-Hägg et al., 2013). ABA was involved in some of these responses. Cuticular wax biosynthesis was ABA-dependent and mediated by MYB94 and MYB96 transcription factors (Lewandowska et al., 2020). A light-induced increase in trichome density and thick leaves was due to high ABA levels in the leaves (Escobar-Bravo et al., 2018). Furthermore, ABA promoted callose synthesis and deposition by negatively regulating callose degrading pathogenesis-related protein 2 (PR2) (Oide et al., 2013). Similarly, ABA up-regulated callose deposition and antiviral RNA silencing mechanism to evade virus attack (Alazem and Lin, 2020).

## Stomatal Closure by Compounds Other Than ABA and Their Interactions During Stomata Closure

Besides ABA, several other compounds increase when plants are exposed to stress, which close stomata and help plant defense responses. These compounds can be grouped into three categories: hormones, elicitors, and metabolites (Table 3). The hormones include MJ, SA, ET, and BRs. MJ was the most effective one and induced stomatal closure by elevating pH, ROS, NO, and Ca^2+^ leading to activation of anion channels, similar to ABA action (Munemasa et al., 2007; Gonugunta et al., 2009; Agurla and Raghavendra, 2016). Further studies in detail are needed to understand the effects of ET as well as BRs on closure.

**TABLE 3:** A spectrum of hormones (other than ABA)/elicitors/PAMPs and other metabolites capable of inducing stomatal closure and the basis of their action.

Compound	Effect on stomata	Plant	References
Allyl isothiocyanate (AITC)	Produces ROS and NO and elevates of cytosolic Ca^2+^	*Arabidopsis thaliana*	Khokon M.A. et al., 2011
β-aminobutyric acid (BABA)	Triggers ABA accumulation under drought	*Triticum aestivum*	Du et al., 2012
Cerato-platanin (CP)	Produces ROS and closes stomata	*A. thaliana*	Baccelli et al., 2014
Chitin oligosaccharide (CTOS)	Elevates Ca^2+^ and activates SLAC1	*A. thaliana*	Ye et al., 2020b
Chitosan	Mediates the production of NO, ROS and Ca^2+^ levels	*Pisum sativum*	Srivastava et al., 2009
Cryptogein	Increases the levels of ROS and NO	*A. thaliana*	Gayatri et al., 2017
γ-aminobutyric acid (GABA)	Represses 14-3-3 proteins and influx of anions into the vacuole	*A. thaliana*	Mekonnen et al., 2016
Flagellin22 (flg22)	Accumulates ROS and activates SLAC	*A. thaliana*	Deger et al., 2015
Harpin	Increases the levels of ROS and NO	*A. thaliana*	Gayatri et al., 2017
Lipopolysaccharide (LPS)	Activates NOS and produces NO in guard cells	*A. thaliana*	Melotto et al., 2006
Methyl jasmonate (MJ)	Promotes H_2_O_2_ production and cytosolic alkalinization	*A. thaliana*	Suhita et al., 2004
Oligogalacturonic acid (OGA)	Increases cytosolic Ca^2+^ and ROS levels	*Lycopersicon esculentum*	Lee et al., 1999
PAMP induced peptide 1 (PIP1)	Activates Ca^2+^ channels and S-type anion channels	*A. thaliana*	Shen et al., 2020
Salicylic acid (SA)	Induce production of ROS, NO and cytosolic Ca^2+^	*A. thaliana*	Khokon M.A. et al., 2011
Yeast elicitor (YEL)	Produces ROS and NO production	*A. thaliana*	Khokon et al., 2010

*The abbreviations are listed in the Appendix (last page).*

Salicylic acid (SA) is considered a plant defense hormone with overlapping functions as an elicitor (Ding and Ding, 2020). SA-induced stomatal closure was mediated by ROS produced primarily through peroxidase (not NADPH oxidase, as in ABA). The other downstream events of NO production and ion channel modulation in guard cells were similar to ABA’s action (Hao et al., 2010; Khokon A.R. et al., 2011; Khokon et al., 2017; Wang et al., 2018). Thus, there is an overlapping of signaling pathways mediated by SA and ABA to cause stomatal closure in Arabidopsis. Several microbial elicitors (chitosan, flg22, harpin, and cryptogein) promoted stomatal closure and prevented pathogens’ entry. These elicitors produce NO and ROS via nitric oxide synthase (NOS) and NADPH oxidase, respectively (Klüsener et al., 2002; Melotto et al., 2006; Agurla and Raghavendra, 2016; Gayatri et al., 2017; Prodhan et al., 2020). The combined action of ROS and NO could be imparting pathogen resistance.

Allyl isothiocyanate (AITC), proline, and PAs are examples of metabolites that accumulate under stress. Despite accumulation in large quantities, proline, a compatible osmolyte, caused only a partial closure (Raghavendra and Reddy, 1987). PAs (including putrescine, spermidine, and spermine) accumulated during water stress and pathogen attack (Alcázar et al., 2010; Hatmi et al., 2018). The oxidation of PAs by polyamine oxidase raised ROS levels, followed by NO to cause stomatal closure similar to ABA (Agurla et al., 2018b). Similarly, AITC promoted stomatal closure and defense responses against biotic components (Khokon M.A. et al., 2011; Ye et al., 2020a).

The hormones, elicitors, and metabolites described above interact markedly with ABA and act in tandem to promote abiotic stress tolerance (Table 4). ABA’s interactions with SA or MJ to work together during stomatal closure and pathogen resistance are well-known (Koo et al., 2020; Wang J. et al., 2020). For e. g., MJ promoted ABA biosynthesis by inducing the AtNCED3 gene expression in Arabidopsis (Hossain et al., 2011). ABA was needed during SA-action on stomata (Wang et al., 2018). Conversely, elevated ABA triggered SA biosynthesis by activating SID2 and promoted stomatal closure (Prodhan et al., 2018). These reports confirm the synergy between MJ, SA, and ABA during stomatal closure.

**TABLE 4:** Interaction of ABA with other compounds during the stress responses that induce stomatal closure.

Hormone / Compound	Interaction with ABA	Stress / or HR response	Plant	References
**Hormones**				
Methyl-Jasmonate (MJ)	Common signaling components with ABA action	Drought	*Arabidopsis thaliana*	Suhita et al., 2004
Ethylene (ET)	Interacts with ABA signaling and inhibits stomatal closure	Drought	*A. thaliana*	Tanaka et al., 2005
Salicylic acid (SA)	Increases ABA signaling via MAPKs and CPKs	Abiotic/biotic stress	*A. thaliana*	Wang H.Q. et al., 2020
Brassinolide (active BR)	Promote and inhibit ABA-mediated stomatal closure	Drought	*A. thaliana*	Ha et al., 2016
**Elicitors/PAMPs**				
Chitosan	Rise in NO, ROS and cytosolic free Ca^2+^, as in case of ABA	External elicitor application	*Pisum sativum*	Srivastava et al., 2009; Gonugunta et al., 2009
Yeast elicitor (YEL)	Regulates ABA action through MPK9 and MPK12 positively regulate ABA	External elicitor application	*A. thaliana*	Salam et al., 2013
12-oxo-phytodienoic acid (12-OPDA)	Functions together with ABA and controls stomatal aperture	Drought	*A. thaliana*	Savchenko et al., 2014
Flagellin	Merge with ABA at OST1kinase and activates SLAC1 and SLAH3	Bacterial infection	*A. thaliana*	Deger et al., 2015
Harpin	signaling merge at RBOH and induces ROS production	External application	*Nicotiana benthamiana*	Zhang et al., 2009
Lipopolysaccharide (LPS)	Produces rapid NO production via NOS	External application	*A. thaliana*	Melotto et al., 2006
**Other factors**				
Polyamines (PAs)	ABA increases PA biosynthesis	Polyethylene glycol-induced osmotic stress	*Triticum aestivum*	Pál et al., 2018
Strigolactone (SL)	Integrates with ABA signaling and enhances stomatal closure	Drought	*Oryza sativa*	Haider et al., 2018
Carbon dioxide (CO_2_)	Enhances ABA signaling via MPKs	Elevated CO_2_	*A. thaliana*	Tõldsepp et al., 2018
Trehalose	Enhanced drought tolerance and ABA signaling	Drought	*Solanum lycopersicum*	Yu et al., 2019
**Signaling components**				
Cytosolic free Ca^2+^	Cross-talk with H_2_O_2_ and NO during ABA action	Extra cellular Ca^2+^	*A. thaliana*	Wang et al., 2012
Phospholipase D (PLDα and PLDδ)	Mediates ABA signaling in guard cells	Multiple abiotic stresses	*A. thaliana*	Uraji et al., 2012
Hydrogen sulfide (H_2_S)	Regulates ABA signaling by persulfidation OST 1	Drought	*A. thaliana*	Chen S. et al., 2020
Phosphatidic acid (PA)	Gα subunit mediates ABA action in response to PA	Drought	*A. thaliana*	Mishra et al., 2006

*The abbreviations are listed in the Appendix (last page).*

An SA-receptor, NPR1, mediated chitosan signaling in guard cells (Prodhan et al., 2020). SA, chitosan, and ABA interacted during stomatal closure by activating MAP kinases (MPK9 and MPK12) (Salam et al., 2012; Khokon et al., 2017). Elevated levels of PAs stimulated biosynthesis of ABA (Yamasaki and Cohen, 2006; Alcázar et al., 2010). In turn, ABA stimulated oxidation of Pas to elevate H_2_O_2_ and NO, and stomatal closure (An et al., 2008; Konstantinos et al., 2010). Such interactions could fine-tune ABA’s effects to strengthen the plant defense reactions against both abiotic and biotic stresses. The direct role of PAs and proline in pathogen resistance is not clear.

## Countermeasures by Pathogens

The stomatal closure by ABA cannot be a permanent strategy to prevent microbial entry, as pathogens, such as Puccinia, can enter leaves through places other than stomata (Mendgen et al., 1996; Solanki et al., 2019). Therefore, we do not mean to overemphasize the role of ABA-induced stomatal closure as the sole mode of adaptation. Also, stomata need to open subsequently to keep up the gas exchange and normal plant function. At the same time, microbial pathogens initiate counteractive measures to reopen stomata, by either effectors, (such as coronatine or fusicoccin, Schulze-Lefert and Robatzek, 2006; Gudesblat et al., 2009a; Melotto et al., 2017), locking the open-stomata (Prats et al., 2006) or even killing guard cells to prevent their closure (Ye et al., 2020b).

Some of the pathogens secrete a cocktail of cell wall digesting enzymes to facilitate the entry through the epidermis into the leaves (Mendgen et al., 1996). The pathogens can also restrict the biosynthesis/actions of ABA and related hormones (Zeng et al., 2010; Robert-Seilaniantz et al., 2011). Further, pathogens too can trigger programmed cell death (PCD) of host tissue facilitating the spread of infection (Hofius et al., 2017; Huysmans et al., 2017). Despite the counteractive efforts by pathogens, ABA can still contribute to plant defense. It is known that ABA could initiate multifaceted measures involving hypersensitive response (HR) and long-term adaptation on its own or by synergetic interaction with other hormones, such as SA or MJ, to ensure improved resistance (described below).

## ABA-Interaction With Gasotransmitters

The role of gasotransmitters in stomatal regulation requires special mention. In addition to NO, two more gaseous signaling molecules (gasotransmitters), hydrogen sulfide (H_2_S), and carbon monoxide (CO) produced within plant cells are an integral part of ABA-dependent stomatal closure as well as other stress conditions. These three gasotransmitters interacted with ABA-signaling during drought (García-Mata and Lamattina, 2013; Yao et al., 2019; Gahir et al., 2020). Under abiotic stress, ABA could elevate the levels of NO as well as CO or H_2_S. For e.g., ABA activated heme oxygenase (HO), thereby increased CO levels and caused stomatal closure (Cao et al., 2007; Wang and Liao, 2016). In turn, NO elevated the levels of H_2_S by regulating H_2_S producing enzymes (L/D-cysteine desulfhydrases) (Kolupaev et al., 2019; Gahir et al., 2020). Similarly, CO promoted both NO and ROS synthesis, facilitating stomatal closure during abiotic stress (Song et al., 2008; He and He, 2014). Thus, a triangular interaction appears to be operating in guard cells. These interactions and synergistic actions need to be examined further. We, however, feel that among the three gasotransmitters, NO could be the significant signaling molecule. Like in the case of ROS, the production of NO can also be triggered by microbial pathogens to activate defense-related genes (e.g., phenylalanine ammonia-lyase and pathogenesis-related protein-1) that play a significant role in acquired pathogen resistance (Romero-Puertas et al., 2004; Ma and Berkowitz, 2016). NO produced in response to lipopolysaccharide contributed towards resistance against *Pst* DC3000 (Melotto et al., 2006). The upregulation of H_2_S production suggested a strong association between H_2_S and plant defense (Shi et al., 2015; Gahir et al., 2020). Further studies on these protective abilities of gasotransmitters to improve pathogen resistance could help achieve plants’ resilience.

Gasotransmitters exert their actions by mediating post-translational modifications (PTMs) such as S-nitrosylation, nitridation, and persulfidation of target proteins (Scuffi et al., 2016; Kolupaev et al., 2019; Gahir et al., 2020). These PTMs seem to exert different effects. Accumulation of H_2_S by ABA mediates the persulfidation of SnRK2.6 to promote stomatal closure by ABA (Chen S. et al., 2020). S-nitrosylation, mediated by NO, inhibited OST1/SnRK2.6 kinase activity and limited stomatal closure (Fancy et al., 2017). Detailed experiments on such contrasting effects of NO and H_2_S would unravel the mechanism of interaction between gasotransmitters and ABA during stomatal closure and plant defense against pathogens.

## Signaling Components in Guard Cells Triggered by ABA: Role in Stomatal Closure and Pathogen Resistance

Stomatal closure is the result of turgor loss in guard cells because of increased cation/anion efflux. A well-defined transduction pathway mediates the events during stomatal closure by ABA or other compounds, as illustrated in Figure 1. Binding of ABA to its receptor inactivates protein phosphatase 2C resulting in the activation of OST1 kinase, which stimulates NADPH oxidase (due to phosphorylation) enzyme to generate ROS and then the production of NO. Both ROS and NO can elevate levels of cytosolic Ca^2+^. The high levels of ROS, NO, and Ca^2+^ act either directly or together to activate anion/cation efflux channels while inhibiting the influx channels. The final result is the loss of cations/anions from guard cells, resulting in turgor loss and stomatal closure (Agurla et al., 2018a). These three secondary messengers (ROS, NO and Ca^2+^) can also stimulate the production of other signaling components such as phospholipase C, phospholipase D, phosphatidic acid, and inositol 1,4,5-triphosphate besides raising cytosolic pH, all contributing to stomatal closure. Apart from well-known NO, other gasotransmitters, i.e., CO and H_2_S, are also involved in ABA-induced stomatal closure.

In recent years, another signaling component, reactive carbonyl species (RCS) was found to play a significant role in stomatal closure. These RCS are products of lipid oxidation, produced and scavenged during various developmental processes, including PCD (Biswas and Mano, 2015). Montillet et al. (2013) suggested that RCS (also called oxylipins) played a dominant role during stomatal closure by biotic factors (e.g., elicitors from pathogens), compared to ROS during the action of ABA (typical of abiotic stress factor). Soon, detailed reports appeared that RCS could function downstream of ROS production during closure by ABA and MJ in *Nicotiana tabacum* and *Arabidopsis thaliana* (Islam et al., 2016, 2019; Islam M.M. et al., 2020). Recently, RCS was found to activate CPK6, promote the elevation of Ca^2+^ and activate SLAC1, leading to stomatal closure (Islam M.M. et al., 2020). These observations imply that RCS and ABA could enable guard cells to respond to both biotic and abiotic stress conditions. Several authors had reviewed the details of the ABA-induced signal transduction pathway (Raghavendra et al., 2010; Munemasa et al., 2015; Sierla et al., 2016; Agurla et al., 2018a; Kolbert et al., 2019; Saito and Uozumi, 2019; Sun et al., 2019).

Several of the signaling components during ABA-induced stomatal closure can protect against pathogens (Table 2). The three major secondary messengers, triggered by ABA (namely ROS, NO, and Ca^2+^) can initiate defense processes such as stomatal closure and PCD (Sewelam et al., 2016; Suzuki and Katano, 2018). ABA-induced NO can act as a signaling molecule to initiate adaptive responses against abiotic (UV, drought, and salinity) or biotic factors (pathogens or elicitors). The reaction products of ROS and NO (like peroxynitrite) and NO-mediated post-translational modifications can all act together to initiate defense responses (Bellin et al., 2013; León et al., 2014; Arnaud and Hwang, 2015). The rise in cytosolic Ca^2+^ was often required to induce HR as a plant immunity response (e.g., against microbial pathogens). Other compounds involved in ABA signaling, like phospholipase D and phosphatidic acid, were also associated with plants’ defense against pathogens (Li and Wang, 2019; Moeder et al., 2019). The ability of H_2_S, a gasotransmitter, to impart resistance against typical plant pathogen (*Pseudomonas syringae*) suggests a link between stomatal closure and adaptation to plant pathogens (Shi et al., 2015; Gahir et al., 2020). The promotion or inhibition of ROS and NO production by gasotransmitters can be a significant factor during plant defense.

*Arabidopsis thaliana* had been an excellent model to study and validate the components/mechanisms of plant function. Several mutants of *A. thaliana* were employed to establish ABA’s signaling components (Table 5). These mutants fall under three groups: those with altered ABA biosynthesis/reception or deficient in signaling compounds or those with altered stomatal response independent of ABA. The mutants who cannot close their stomata also lose their ability to resist pathogens, becoming hypersensitive to pathogens. These observations emphasize the strong association of stomatal closure by ABA or related compounds with altered pathogen resistance.

**TABLE 5:** List of Arabidopsis mutants deficient in ABA biosynthesis/signaling pathway and their susceptibility to pathogen attack.

Mutant	Deficiency	Response to pathogens	References
**ABA biosynthesis / Reception**			
*aao3* and *aba2* (Abscisic aldehyde oxidase)	Impaired ABA biosynthesis	Susceptible to *Pythium irregulare*	Adie et al., 2007
*aba3* (Abscisic acid)	Abscisic acid biosynthesis	Neither flg22 nor lipopolysaccharide (LPS), failed to induce closure	Melotto et al., 2006
*ataf1* (NAC protein)	Increased ABA levels	Reduced resistance to biotrophic fungus	Wu et al., 2009
*abi1* and *abi2* (ABA insensitive)	PP2C, needed for ABA signaling	Stomatal closure is absent in response to *Trichoderma* species	Contreras-Cornejo et al., 2015
*coi1* (Coronatine-insensitive)	MeJA-induced stomatal closure	COR not able to prevent ABA-induced stomatal closure	Melotto et al., 2006
**ABA-signaling components**			
*agb1* (Arabidopis G β-subunit)	G-protein β subunit, involved in GTPase activity	Stomata remained open and highly susceptible to *Pseudomonas* sps	Lee et al., 2013
*cpk3-2 cpk6-1*	Ca^2+^-dependent protein kinases	SA and ABA-induced stomatal closure is impaired	Prodhan et al., 2018
*gcn2* (General control non-derepressible 2)	GCN kinase activity	Less effective in closing stomata and resisting *P. syringae*	Liu et al., 2019
*lcbk1*(Long-chain base kinase1)	Long-chain base kinase1	Susceptible to virulent pathogens and not able to close stomata	Gupta et al., 2020
*mpk3 mpk6* and *mkk4 mkk5*	Mitogen-activated protein kinases	Unable to close stomata in response to PAMP or *Pst*	Su et al., 2017
*ost1* (Open stomata1)	Reduced K^+^efflux	Stomatal closure is impaired in response to flg22	Deger et al., 2015
*ost2* (open stomata2)	H^+^-ATPase	ABA-insensitive and flg22/LPS unable to induce stomatal closure	Liu et al., 2009
*rbohD* (Respiratory burst oxidase homologue)	Impaired ROS production	Impaired flg22 responses and stomatal closure	Kadota et al., 2014
*slac1*(Slow anion channel 1)	Slow anion channel	Hyposensitive to *Pst* and reduced stomatal closure	Shen et al., 2020
**Other effectors**			
*era1* (Enhanced response to ABA1)	Farnesyl transferase β subunit	Hypersensitive to ABA and virulent pathogens	Jalakas et al., 2017
*eds1* (Enhanced Disease Susceptibility)	Salicylic acid biosynthesis	Ability to close stomata in response to bacteria and LPS is compromised	Melotto et al., 2006
*lox1* (Lipoxygenase)	Lipoxygenase activity	Compromised ability to close stomata in response to virulent and avirulent pathogens	Montillet et al., 2013
*pip2;1* (Plasma membrane intrinsic protein)	Plasma membrane aquaporin	Impaired stomatal closure in response to ABA and flg22	Rodrigues et al., 2017
*rpfF* and *rpfC* (Regulation of pathogenicity factor)	Synthesis and perception of diffusible molecule	Stomatal closure is absent in response to *Xcc* and *Pst*	Gudesblat et al., 2009b

*A few other instances of mutants altered in stomatal closure and pathogen sensitivity are also included. The abbreviations are listed in the Appendix (last page).*

### Other Points to Be Considered

Evidence is emerging that ABA may not always impart resistance but increase plants’ susceptibility to abiotic or biotic factors by compromising defense responses (Gietler et al., 2020). For e.g., ABA can act differently depending on the pathogen status: pre-entry or post-entry phases. During the early stages, induction of stomatal closure along with stimulation of wax and callose synthesis could reinforce the plant’s defense. However, in the post-invasion stage, ABA can be antagonistic and increase susceptibility to microbes (Alazem and Lin, 2015; Arnaud and Hwang, 2015). Accumulation of ABA at infection site repressed events involving disease resistance against *Cercospora* in beetroot (Schmidt et al., 2008). Elevated ABA levels promoted sugar transport to fungi and enhanced the infection of wheat by *Puccinia striiformis* sp. *tritici* (pst) (Huai et al., 2019). Furthermore, increased ABA levels antagonized plant’s defense responses by suppressing SA or MJ induced defense gene expression, callose deposition, and basal resistance against *Fusarium oxysporum* or *Magnaporthe grisea* (Anderson et al., 2004; Jiang et al., 2010; Lim et al., 2015; Ulferts et al., 2015).

We believe that ABA could play a significant role in restricting or at least delaying the pathogen entry and subsequent infections, at least in the case of bacteria (Table 5 and description below). We acknowledge that the role of stomata should not be generalized, as the experiments done on pathogen infection with Arabidopsis (non-host) may not be all applicable with typical host species, such as wheat or barley.

## Subsequent Effects of ABA Besides Stomatal Closure Towards Adaptive Responses: Pre-Entry and Post-Entry Phenomena

Pathogen resistance cannot be entirely due to stomatal closure, and the action of ABA needs to continue beyond stomata. Often during infection, an increase in ABA levels led to multiple events that help against abiotic stress and disease resistance (Shafiei et al., 2007). Even if pathogens manage to enter the intercellular spaces, elevated ABA can initiate a spectrum of events that restrict the multiplication and spread of plant pathogens inside leaves. For e.g., ABA can raise the levels of ROS, NO, and Ca^2+^ (Wendehenne et al., 2004; Moeder et al., 2019; Sadhu et al., 2019). An important consequence of elevated ROS, NO, and Ca^2+^ is HR response, leading to PCD in several crop species while protecting against pathogens. The HR may also include callose deposition in cell walls, increased cuticular biosynthesis (Luna et al., 2011; Lewandowska et al., 2020), and blocking of plasmodesmata (Huysmans et al., 2017). Further, the modulation by ABA of miRNA can restrict viral replication and the viral movement due to blocked plasmodesmata, thus causing antiviral silencing (Staiger et al., 2013; Alazem and Lin, 2017).

**FIGURE 2:**
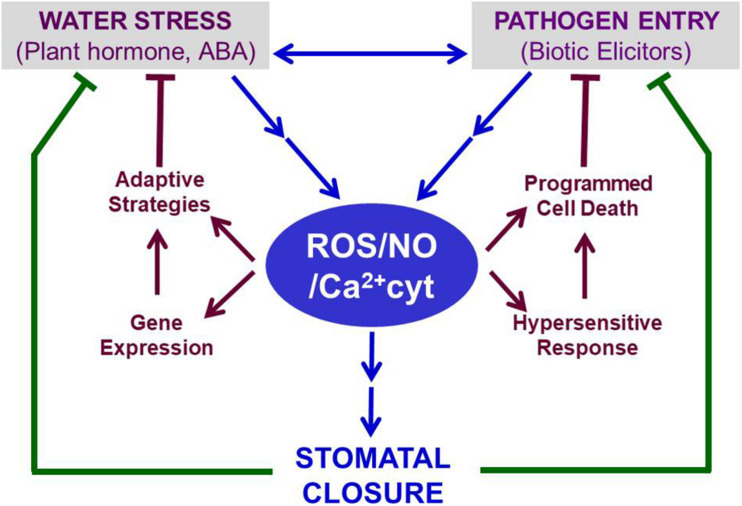
Stomatal closure induced under conditions of abiotic (e.g., drought) or biotic (e.g., pathogens) stress serves as a common defense mechanism. In guard cells, ABA typically raises the levels of ROS, NO, and Ca^2+^. These three secondary messengers bring out stomatal closure through a series of signaling events (as illustrated in Figure 1). The retention of water within leaves, when stomata are closed, helps to relieve water stress. In parallel, the closed stomata restrict microbial pathogens’ entry into leaves. The trio of ROS, NO, and Ca^2+^ parallelly induce adaptive events to mitigate water stress and limit pathogen spread by triggering HR and PCD. Thus, ROS, NO, and Ca^2+^ can be considered vital regulators, participating in ABA-induced defense against abiotic and biotic stress. Further details are described in the text. Abbreviations used here are listed in the Appendix.

Other ABA-promoted events include the activation of genes involved in either accumulation of compatible solutes for osmotic adaptation or PCD to restrict the spread of pathogens within leaves or enhanced secondary metabolites production (Figure 2). For example, ABRE-binding proteins (AREBs) and ABRE-binding factors (ABFs) up-regulate stress-responsive genes, involved in short-term and long term adaptations to abiotic stresses, including drought, cold, and heat (Verma et al., 2016; Vishwakarma et al., 2017). The accumulation of proline on exposure to water stress or ABA (Stewart, 1980; Planchet et al., 2014) can serve the dual purpose of providing compatible solute for osmotic adjustments in leaves and causing partial stomatal closure in the epidermis (Raghavendra and Reddy, 1987). Elevated proline levels due to ABA can further offer the plant defense against pathogens (Qamar et al., 2015; Christgen and Becker, 2019). There have been claims that proline accumulation is not all ABA-dependent (Savouré et al., 1997). It is not clear if ABA is the master regulator of proline accumulation or a consequence of stomatal closure. ABA and proline’s combined action can be beneficial under hypoxic stress (Cao et al., 2020). Similarly, ABA, proline, and PAs can help together during plant adaptation to osmotic stress (Pál et al., 2018).

Several ABA responses are beneficial during biotic stress, as well. ABA and SA promoted the accumulation of anti-microbial flavan-3-ols, enhancing the plant defense against rust infection in two popular trees, Poplar and Malus (Lu et al., 2017; Ullah et al., 2019). Similarly, the ABA-activated MYC2 transcription factor was essential for defense against *Meloidogyne incognita* (Xu et al., 2019). PCD is a controlled process and is a consequence of high ROS and NO levels typically up-regulated by ABA (Petrov et al., 2015; You and Chan, 2015). In plants, PCD is often an adaptive response during abiotic/biotic stress (Fagundes et al., 2015; Burke et al., 2020). However, PCD can also aggravate plant disease (Huysmans et al., 2017). ABA was involved in the induction of cell death around the wounded site (Cui et al., 2013). Further experiments are needed to establish if the process of PCD is incidentally associated with ABA or if ABA is the causal factor.

Enhanced production of secondary metabolites is another defense mechanism of plants against stress (Kumar and Sharma, 2018; Khare et al., 2020). Abscisic acid itself is a secondary metabolite produced during stress and can play a significant role in secondary metabolite production when plants encounter abiotic or biotic stress factors (Murcia et al., 2017). ABA-induced increase in flavonoids and other metabolites served as a defensive measure against UV-B radiation (Mazid et al., 2011). *Trichoderma harzianum* infection increased ABA levels, which helped in osmotic adaptation under drought by restricting water loss and increasing osmolytes, like proline (Mona et al., 2017). Such interactions between ABA and secondary metabolite production are quite exciting and need to be examined in detail.

## Conclusion

Based on the extensive literature available, we tried to emphasize the role of ABA-induced stomatal closure as an essential component of plant defense against both limited water and pathogens (Figure 2). ABA’s role is complemented further by the cross-talk and interaction of ABA with other hormones, microbial elicitors, and metabolites. We, therefore, emphasize ABA can play either a direct role or an indirect role as well. The stomatal closure by ABA can be considered a quick short-term response. However, the three vital secondary messengers involved in ABA-signaling, namely ROS, NO, and cytosolic free Ca^2+,^ can promote events, such as osmolyte accumulation, up-regulation of adaptive genes, HR and PCD. These events facilitate the long-term adaptation of plants against abiotic stress as well as pathogens. ABA’s ability to induce stomatal closure may not always be due to changes occurring in guard cells’ secondary messengers. For e.g., chitosan, a microbial elicitor, can cause the death of guard cells (Ye et al., 2020b), likely to make them non-functional. This aspect is thought-provoking and needs to be examined further using ABA or other hormones, such as SA or MJ.

There are emerging areas that are related to the role of ABA and stomatal closure in plant defense. ABA’s ability to induce priming could help plants tolerate heat or drought stress occurring later (Zhang et al., 2019; Wang X. et al., 2020). Similarly, plants could have a transcriptional memory of ABA and MJ that can be useful for long-term adaptations (Avramova, 2019). Because of the well-documented importance, there had been recurring attempts to discover ABA-analogs or ABA-agonists. Stomatal closure and signaling components in guard cells can be excellent model systems to monitor such compounds. A few ABA-analogs were found, which mimic ABA to induce stomatal closure (Puli and Raghavendra, 2012; Vaidya et al., 2019). An extension of such work would open up an exciting possibility of exploiting ABA-analogs to improve plants/crops’ water-use efficiency. There were reports that ABA and or stomatal closure may not be crucial, particularly during fungal pathogen infection of crops, e.g., barley and wheat. A few experiments on mutants of such crop plants deficient in ABA or ability to close stomata may help us clarify the exact situation. Plants are indeed known to employ more than one strategy to overcome stress conditions or to optimize metabolism. ABA-induced stomatal closure is one of the approaches, while plants/pathogens could evoke other strategies, as well.

## Author Contributions

AR conceived the idea and developed the outline. All authors reviewed the literature and wrote the article. AR edited the final draft of the review. All authors approved the final version.

## Conflict of Interest

The authors declare that the research was conducted in the absence of any commercial or financial relationships that could be construed as a potential conflict of interest.

## Appendix

**TABLE A1:** List of sentences used in the syntactic awareness task.

12-OPDA,	12-oxo-phytodienoic acid;
AAO,	Abscisic aldehyde oxidase;
ABA,	Abscisic acid;
ABI,	ABA insensitive;
AGB1,	Arabidopsis G β-subunit;
AITC,	Allyl isothiocyanate;
BABA,	β-aminobutyric acid;
COI1,	Coronatine insensitive1;
CPK,	Ca^2+^-dependent protein kinases;
CTOS,	Chitin oligosaccharide;
EDS1,	Enhanced disease susceptibility;
ERA1,	Enhanced response to ABA1;
ET,	Ethylene;
GABA,	γ-aminobutyric acid;
GCN2,	General control non-depressible2;
H_2_S,	Hydrogen sulfide;
IP3,	Inositol 1,4,5-triphosphate;
LCBK1,	Long-chain base kinase1;
LOX,	Lipoxygenase;
LPS,	Lipopolysaccharide;
MJ,	Methyl jasmonate;
MPK,	Mitogen-activated protein kinase;
OGA,	Oligogalacturonic acid;
OST,	Open stomata;
PA,	Phosphatidic acid;
PIP2/1,	PAMP-induced peptide 2/1;
PLD,	Phospholipase D;
RBOH-	Respiratory burst oxidase homologue;
RPF,	Regulation of pathogenicity factor;
S1P,	Sphingosine-1-phosphate;
SA,	Salicylic acid;
SL,	Strigolactone;
SLAC,	Slow anion channel;
YEL,	Yeast elicitor.

## Abbreviations

ABA: Abscisic acid
BRs: Brassinosteroids
CO: Carbon monoxide
ET: Ethylene
Flg22: Flagelling 22
H_2_S: hydrogen sulfide
HR: Hypersensitive response
MJ: Methyl jasmonate
MPK: Mitogen activated protein kinase
NO: Nitric oxide
OST1: Open stomata 1
PAs: Polyamines
PCD: Programmed cell death
RCS: Reactive carbonyl species
ROS: Reactive oxygen species
SA: Salicylic acid
SLAC1: Slow anion channel 1
SLAH3: S-type anion channel 3.
